# Cost of whole genome sequencing for non-typhoidal *Salmonella enterica*

**DOI:** 10.1371/journal.pone.0248561

**Published:** 2021-03-19

**Authors:** Laura Ford, Kathryn Glass, Deborah A. Williamson, Vitali Sintchenko, Jennifer M. B. Robson, Emily Lancsar, Russell Stafford, Martyn D. Kirk

**Affiliations:** 1 Research School of Population Health, The Australian National University, Canberra, Australian Capital Territory, Australia; 2 The Peter Doherty Institute for Infection and Immunity, The University of Melbourne, Melbourne, Victoria, Australia; 3 Marie Bashir Institute for Infectious Diseases and Biosecurity, The University of Sydney, Westmead, New South Wales, Australia; 4 Centre for Infectious Diseases and Microbiology-Public Health, Westmead Hospital, NSW Health Pathology, Sydney, New South Wales, Australia; 5 Sullivan Nicolaides Pathology, Brisbane, Queensland, Australia; 6 Queensland Department of Health, Herston, Queensland, Australia; Health Directorate, LUXEMBOURG

## Abstract

**Background:**

While whole genome sequencing (WGS) may be more expensive than traditional testing and polymerase chain reaction (PCR), simple cost comparisons ignore the potential for WGS to reduce the societal costs of non-typhoidal *Salmonella enterica* through public health action to prevent illness.

**Methods:**

We determined how many cases the use of WGS data would need to prevent to be cost-equal to serotyping and MLVA, or culture independent testing based on PCR in Australia. We then examined the costs and cost-savings of current typing methods compared with WGS in outbreak scenarios.

**Results:**

A median of 275 (90% CrI -55-775) or 1.9% (90% CrI -0.4%-5.4%) of notified serotyped *Salmonella* cases would need to be prevented for WGS to be cost-equal to current typing methods and 1,550 (90% CrI 820–2,725) or 9.6% of all notified *Salmonella* cases would need to be prevented to be cost-equal to PCR. WGS is likely to result in cost savings in prolonged outbreaks, where data can support earlier public health action.

**Conclusions:**

Despite currently having a higher cost per isolate, routine WGS of *Salmonella* was no more expensive than existing typing methods or PCR where >2% of illness was averted.

## Introduction

Culturing human samples for non-typhoidal *Salmonella enterica* informs both clinical diagnosis and public health surveillance. Routine epidemiological typing of cultured *Salmonella* isolates allows health agencies to identify outbreaks and sources of infection, and implement control measures to prevent further illness. Conventional typing methods, such as serotyping, pulse field gel electrophoresis (PFGE), and multiple locus variable-number tandem repeat analysis (MLVA) may differentiate *Salmonella* cases for surveillance and outbreak detection, helping to save lives and reduce costs to human health and industry [1, 2].

New technologies for laboratory testing and typing *Salmonella* are moving in divergent directions. The emergence of culture-independent diagnostic testing through Polymerase Chain Reaction (PCR) assays allows for quick, sensitive and inexpensive *Salmonella* detection, compared with conventional culture on selective media. However, unless reflexive culture (i.e. culture on PCR-positive samples) is performed, no isolate is grown and available for typing. PCR tests alone provide limited information for public health surveillance. This delays the recognition of outbreaks, potentially leading to larger outbreaks and increased societal costs [3].

If an isolate is cultured, whole genome sequencing (WGS) using high-throughput next generation methods has emerged as an alternative to conventional typing methods such as serotyping and MLVA. WGS generates highly discriminatory data on *Salmonella* isolates for surveillance and outbreak detection [4]. WGS has been shown to help detect outbreaks while they are still small and to link food sources to outbreaks, allowing for quick and effective intervention and control [5]. However, at present, public health and laboratory infrastructure required to adopt WGS and sequencing costs can be high.

Although sequencing costs are declining, WGS of foodborne bacterial pathogens is currently still more expensive than PCR and the cost differential with conventional typing methods varies. WGS could reduce the societal costs of *Salmonella* if it can reduce the number of people affected, and the costs of these affected cases are considered. A full economic evaluation is not possible in this context as the effectiveness measure for WGS as a public health intervention is unknown. Therefore, we examined the societal costs of three *Salmonella* testing and typing approaches for *Salmonella* positive samples in terms of how they assist public action to detect outbreaks, implement control measures, and prevent cases in Australia: (1) the current standard of culture, serotyping and MLVA; (2) culture and WGS; and (3) PCR testing.

## Materials and methods

**Fig 1:**
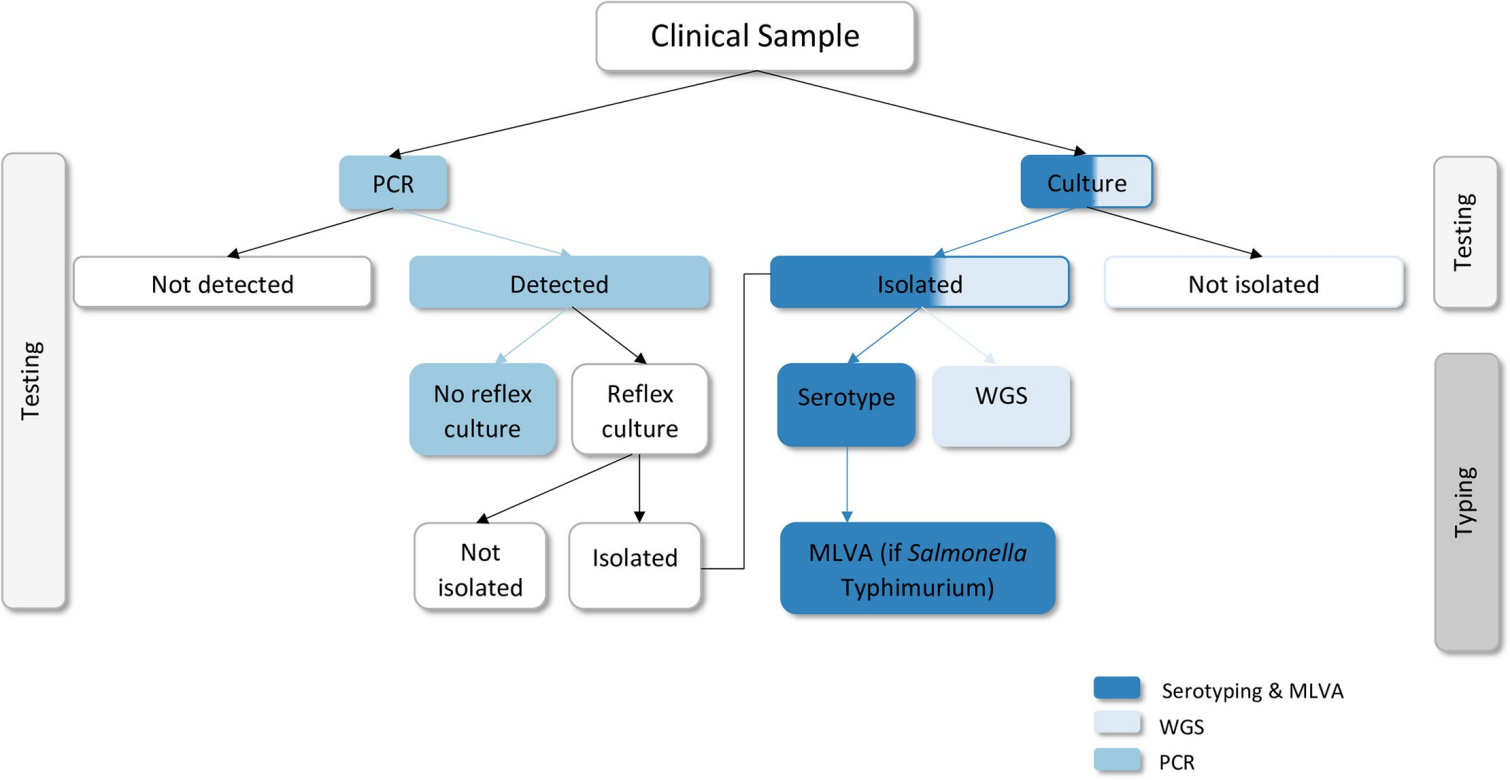
Flow chart of testing and typing methods for *Salmonella* in Australia.

In Australia, once a patient submits a clinical sample to a laboratory, *Salmonella* can either be detected (with PCR) or isolated from that sample. If isolated, several typing methods can be employed to characterise the isolate for public health surveillance and outbreak investigation. Fig 1 explains this process and highlights the three methods for which we have examined costs. We have not costed PCR followed by reflexive culture as this will always be more expensive for *Salmonella*-positive samples than culture and typing. Costs and cost savings are reported in 2018 US dollars (USD), with Australian dollars (AUD) converted to USD using the average monthly exchange rate from Jan-Jun 2018 of 0.7676 from the Reserve Bank of Australia [6] and inflation adjusted for using the Reserve Bank of Australia’s inflation calculator [7]. To assess costs, we first determined how many cases must be prevented for each method to be cost-equal to serotyping and MLVA, and then modelled outbreak scenarios with different intervention points based on test data to assess cost savings.

### Data sources

We used a cost per *Salmonella* case circa 2015 from Ford et al [8] including direct and indirect costs of health care usage, lost productivity, and premature mortality from acute infection, adjusted for inflation to 2018. We adjusted testing costs from the cost per case to enable comparison of the three testing and typing regimes. Cases detected were defined as cases notified to the public health department and *Salmonella* notification numbers from 2017 were obtained from the National Notifiable Diseases Surveillance System (NNDSS) [9]. As 35% of notifications in Australia are serotyped as *S*. Typhimurium (see S1 Appendix) and subsequently undergo multiple variable number tandem repeat analysis (MLVA) [10], we separated *Salmonella* Typhimurium and non-Typhimurium *Salmonella*. We used the mean and range of costs for serotyping, MLVA (*S*. Typhimurium), and WGS (where available) collected from each of the five Australian state public health reference laboratories in mid-2018. For culture and PCR test costs, we calculated cost per test by dividing the total benefits (monetary value spent) by the number of services (number billed) of item reports 69345 and 69496 respectively in the Medicare Benefits Schedule for the financial year 2017/18 [11, 12]. This resulted in a cost of USD 34.70 for PCR and USD 40.61 for culture. To model cost savings in an outbreak scenario, we used simulated data based on Australian *Salmonella* outbreak data [13, 14].

### Analysis

#### Cases prevented

Although not strong enough quantitative evidence to use in a full economic evaluation, there is evidence that use of WGS can enable epidemiologists to detect outbreaks and sources of infection earlier, allowing for quicker public health action to implement control measures and prevent further cases [5]. Therefore, we calculated how many cases WGS data would have to prevent before it would cost the Australian society no more than serotyping and MLVA. As it is difficult to detect outbreaks with PCR, leading to delays in implementing control measures and potentially larger outbreaks [3], we also used the same analysis to calculate how many cases WGS and the status quo would have to prevent before they cost no more than PCR. The equation to determine the threshold is given by:

$$({cost\;per\;test}_{A}+cost\;per\;case)\times {number\;of\;cases\;detected\;with\;test}_{A}=$$

$$({cost\;per\;test}_{B}+cost\;per\;case)\times {number\;of\;cases\;detected\;with\;test}_{B}$$

In addition, to examine the effects of changes in WGS costs, we calculated how much the price would have to drop before WGS was cost equal with (1) serotyping and (2) PCR. We performed these analyses in @Risk version 6 (http://www.palisade.com). Uncertainty intervals for Australia were generated from the minimum and maximum cost estimate for serotyping, MLVA, and WGS from the reference laboratories. We used a PERT distribution for cost estimates with uncertainty to generate median and 90% credible intervals for the number and proportion of cases that need to be prevented, as well as the WGS cost decrease needed, for tests to be cost neutral. Further information is provided in S2 Appendix.

#### Outbreak scenario

The costs of using the testing and typing regimes were examined in three simulated community outbreak scenarios: (1) a point source outbreak, (2) a prolonged outbreak with no peak, and (3) a prolonged outbreak with a late peak. We sourced mean case numbers from Australian *Salmonella* outbreak data [13, 14] to generate random daily case numbers sampled from a Poisson distribution for each outbreak scenario. We estimated the cost of the outbreak using the cost per case and the costs per test as described above. With Microsoft Excel and @Risk, we calculated the cost differences if WGS was used in all three scenarios, and if a product recall or intervention occurred at an earlier time point along the epidemiological curve (30, 60, and 90 days earlier) of the prolonged outbreak scenarios using WGS. While it is unlikely that outbreaks would have the same epidemiological curve if all human samples were only tested by PCR, we have considered outbreak costs using PCR testing in these scenarios for comparison. We compared characteristics of our outbreak scenario models with data on community outbreaks of non-typhoidal salmonellosis from Queensland Health (see S3 Appendix).

## Results

In 2018, Australian reference laboratories reported that the mean cost for serotyping *Salmonella* was USD 42.37 (range USD 13.82–75.22), while MLVA cost USD 52.66 (range USD 24.56–95.95) and WGS cost USD 83.15 (range USD 72.92–95.95). The cost per case of *Salmonella* spp. infection was USD 1,098 (90% CrI USD 623–1,963).

We estimated that approximately 365 (90% CrI 145–775) or 4.2% (90% CrI 2.2%-7.6%) of non-Typhimurium *Salmonella* spp. cases needed to be prevented in Australia in a year for WGS to be cost-equal to serotyping, at current costs (Table 1). Serotyping and MLVA for *S*. Typhimurium was more expensive than WGS, meaning that WGS is already cheaper than current methods for *S*. Typhimurium.

**Table 1:** Number of cases that need to be prevented for WGS and PCR methods to be cost-equal to current methods, Australia, 2018.

	*Salmonella* Typhimurium		Non-Typhimurium *Salmonella*	
	Median cases (90% CrI)	% (90% CrI)	Median cases (90% CrI)	% (90% CrI)
**WGS vs current methods**^**a**^	-80 (-325-100)	-1.4 (-5.6–1.7)	365 (145–775)	4.2 (2.2–7.6)
**Current methods**^**a**^ **vs PCR**	390 (170–790)	6·8 (3.0–13.9)	80 (-115-340)	0·9 (-1.3–3.9)

^a^Current methods are serotyping and MLVA (if serotyped as *Salmonella* Typhimurium).

Combining *S*. Typhimurium and non-Typhimurium cases, 275 (90% CrI -55-775) or 1.9% (90% CrI -0.4%-5.4%) of all notified serotyped *Salmonella* cases needed to be prevented for WGS to be cost-equal to serotyping and MLVA. For WGS to be cost-equal to PCR, 1,550 (90% CrI 820–2,725) or 9.6% (90% CrI 5.1%-17%) of *Salmonella* spp. cases need to be prevented in a year.

If costs for culture and WGS dropped a median of USD 40.65 (95% CrI USD 20.00–60.73), or approximately 33%, then WGS would be cost-equal to culture and serotyping with current case numbers. The costs of culture and WGS would need to drop by a median of USD 89.39 (95% CrI USD 82.53–96.79), or approximately 72% to be cost-equal to PCR with current case numbers.

### Outbreak scenarios

#### Point source outbreak

**Fig 2:**
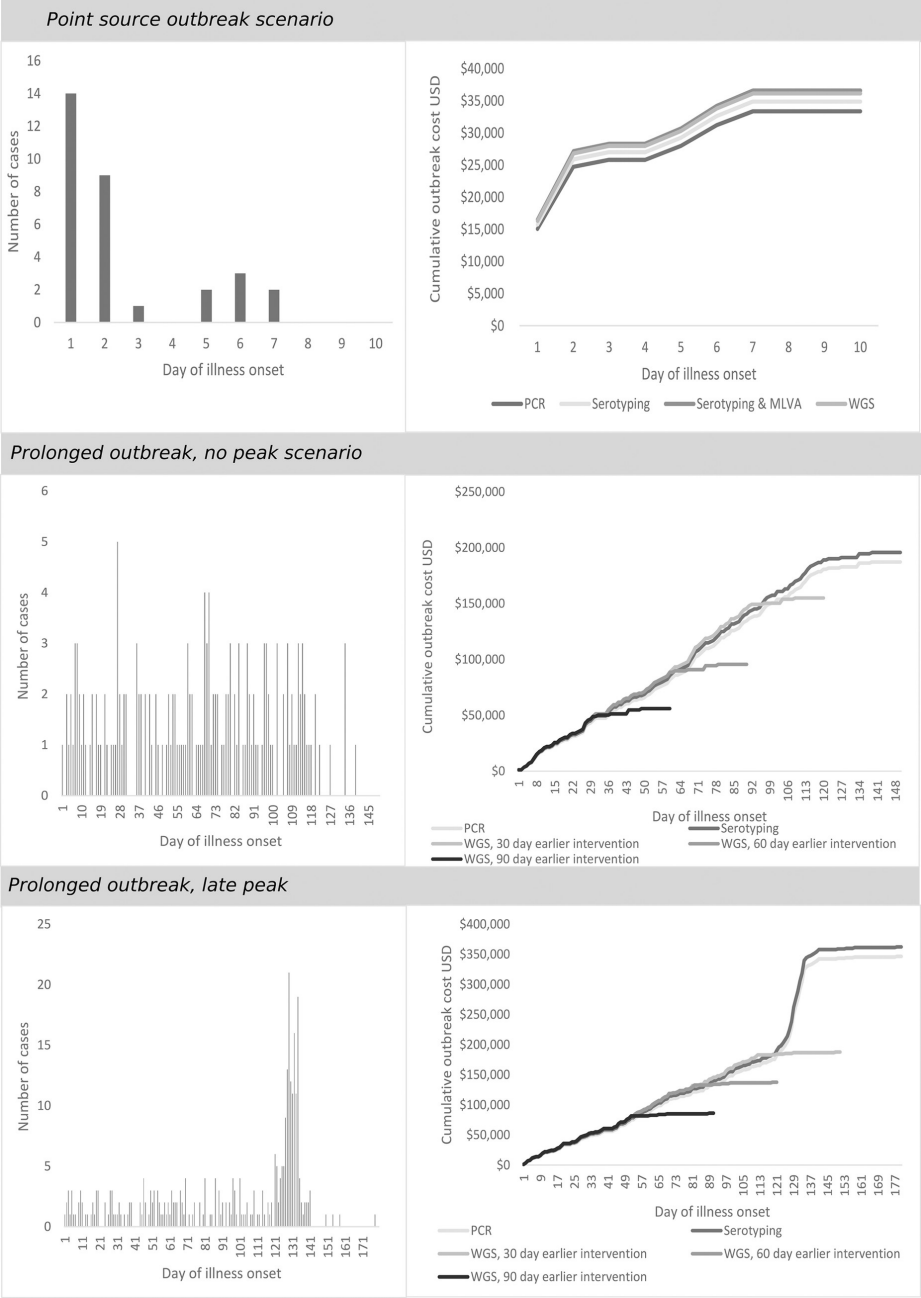
Epidemiological curve and cumulative outbreak case costs in simulated point-source outbreak. Confidence intervals omitted for visual clarity.

As exposure during a point source salmonellosis outbreak tends to occur over a relatively short period (e.g. 1 meal), we used a conservative assumption that interventions will have no effect on reducing case numbers in that outbreak. Therefore, detecting the outbreak sooner through WGS would have no effect on reducing those outbreak costs. In our point source outbreak scenario, there were 31 cases, with illness onsets occurring over 7 days (Fig 2). We estimated the cumulative costs of the outbreak with PCR at USD 35,102 (90% CrI 20,222–61,847), culture and serotyping at USD 36,648 (90% CrI 21,750–63,489), with WGS at USD 37,851 (90% CrI 23,019–64,699) (USD 1,203 more than culture and serotyping), and with culture, serotyping, and MLVA at USD 38,369 (90% CrI 23,416–65,082) (USD 518 more than WGS) (Fig 2).

#### Prolonged outbreak, no peak

We simulated a prolonged salmonellosis outbreak lasting 150 days, with a mean daily case number of 1.5 cases for the first 120 days, and assuming an intervention was put in place at 120 days with a mean daily case number of 0.2 cases for the last 30 days. In this outbreak scenario, there were 174 cases, with a cumulative cost of USD 205,808 (90% CrI 121,842–352,912) using culture and serotyping, USD 212,807 (90% CrI 129,134–360,199) using WGS (Fig 2), and USD 215,587 (90% CrI 131,564–362,438) using culture, serotyping and MLVA. While an outbreak like this may not be detected if all samples were PCR-only, if it were detected with the same epidemiological curve, we estimated a cumulative cost of USD 197,117 (113,315–344,546) using PCR (Fig 2). If WGS enabled early detection such that the intervention was put in place 30 days earlier, this would result in a savings of USD 34,359 (90% CrI 14,606–68,792) over PCR or USD 42,814 (90% CrI 22,756–77,399) over culture and serotyping, rising to a savings of USD 97,112 (90% CrI 52,167–174,489) over PCR or USD 105,057 (90% CrI 60,323–183,673) over culture and serotyping if the intervention was 60 days earlier, and USD 138,783 (90% CrI 77,197–244,690) over PCR or USD 146,711 (90% CrI 85,408–254,676) over culture and serotyping if the intervention was 90 days earlier (Fig 2).

#### Prolonged outbreak, late peak

We simulated a prolonged salmonellosis outbreak based on real outbreak data lasting 180 days, with a 1-week peak occurring approximately 120 days after the start of the outbreak [14]. We assumed a mean daily case number of 1.35 cases for the first 120 days, 4.5 cases for 7 days, 13.23 cases for 7 days, 2.25 cases for 7 days, and 0.14 cases for the last 39 days. We assumed an intervention was put in place at 140 days, following the peak. In this outbreak scenario, there were 322 cases, with a cumulative cost USD 329,186 (90% CrI 194,382–575,442) using culture and serotyping, USD 340,759 (90% CrI 206,014–586,903) using WGS (Fig 2), and USD 344,359 (90% CrI 209,629–590,760) using culture, serotyping and MLVA. Again, while an outbreak like this may not be detected if all samples were PCR-only, with the same epidemiological curve, we estimated a cumulative cost of USD 315,846 (90% CrI 181,015–561,523) using PCR (Fig 2). If WGS were able to detect the outbreak or food vehicle earlier, this would result in a savings of USD 119,224 (90% CrI 62,199–223,053) over PCR or USD 132,739 (90% CrI over culture and serotyping if the intervention as put in place 30 days earlier, USD 171,771 (90% CrI 93,859–313,571) over PCR or USD 185,042 (90% CrI 107,353–326,844) over culture and serotyping if the intervention was put in place 60 days earlier, and USD 225,469 (90% CrI 126,201–405,909) over PCR or USD 238,848 (90% CrI 139,689–419,376) over culture and serotyping if the intervention was put in place 90 days earlier (Fig 2).

## Discussion

In this study, we examined costs of three *Salmonella* testing and typing systems in and their relevance to public health actions. We used Australian data from 2018 as a representative example of a country with a relatively low incidence of non-typhoidal salmonellosis and established systems of public health laboratory surveillance for foodborne diseases. Our findings demonstrated that WGS data needs to prevent approximately 2% of all notifications currently serotyped, or approximately 10% of all notifications if they were only tested through PCR to be cost-equal to current testing and typing methods and PCR, respectively. WGS could also significantly reduce costs in prolonged outbreaks where the data helps public health officials to implement interventions earlier. Even in point source outbreaks, where WGS is potentially least effective at reducing costs, by linking multiple point source outbreaks or linking a specific food or food preparation practice to illness, WGS data could help to avert future cases and reduce costs. Our findings present a compelling case for widespread adoption of WGS in public health reference laboratories in Australia for *Salmonella* surveillance and investigation.

As there are few examples in the literature of prospective use of WGS for surveillance and outbreak detection, the effectiveness of preventing foodborne illness is largely unknown and the average time from outbreak detection to the implementation of control measures has not been well measured. However, there is evidence that WGS data is more sensitive and specific in linking salmonellosis cases than current *Salmonella* typing methods. It also helps link cases over wide geographical areas and longtime frames, reveals geographically distinct clusters, differentiates cases not in an outbreak, results in better detection of *Salmonella* in food sources and faster source tracking, and can provide evidence for the implementation of *Salmonella* control plans in the food industry [15–24]. All of these advancements could lead to earlier intervention and support more targeted use of public health resources, therefore reducing costs. In the USA, PulseNet, a molecular subtyping network of federal, state, and local public health laboratories has demonstrated significant economic and public health benefits through averting foodborne illness with PFGE [2], benefits which will likely be increased through the use of WGS. A Canadian study estimated that WGS will result in a net benefit of $5.21 million for reported salmonellosis cases, $64·98 million if there is a 50% reduction of illness, or $90·25 million if there is a 70% reduction in illness through the reduction of direct and indirect salmonellosis costs from contamination in fresh produce, poultry, and eggs [25].

We have not addressed the costs of PCR and reflexive culture for all human samples in this study, instead examining only costs associated with *Salmonella* positive samples first detected either by PCR or culture and the impact on public health action. While PCR-only testing remains the cheapest option at present in terms of raw costs, it results in a loss of serotyping and subtyping capability [3], making outbreak detection, linking human *Salmonella* infections to specific sources and public health follow-up impossible. Therefore, while we estimated PCR costs in our outbreak scenarios, it is likely that outbreak epidemiological curves would be much larger, resulting in higher costs over other testing and typing methods. In Australia, the large majority of human disease due *Salmonella* are still confirmed by culture, either in the first instance or reflexively. Since the introduction of the multiplex PCR test in late 2013, the proportion of notifications without a serotype has increased from around 2% annually (2009–2012) to 10% in 2017 [9]. Although some of these may have been PCR detections that were not reflexively cultured, it may also be a reflection of the increased sensitivity of PCR over culture [26]. A study in one Australian state found that in 2014, while 6% of *Salmonella* notifications were diagnosed only by PCR test, a further 12% were PCR positive, but culture negative [27]. While reflexive culture represents an additional cost for pathology providers, it is possible that through the increased sensitivity of PCR, there are more isolates for further typing, assisting outbreak investigations.

Several limitations of this study have to be acknowledged. First, only per-isolate sequencing costs were taken into account; we did not considered laboratory expenditures associated with the transition to WGS. However, WGS costs have declined over time [28] and this trend will likely continue as WGS replaces existing typing techniques for foodborne pathogens. The *Salmonella* case cost estimate used here focuses on healthcare expenditure and lost productivity, and does not include costs of *Salmonella* for industry. The rapid detection of outbreaks can reduce costs for industry and trade, which we have not accounted for in this study. We have not conducted a full economic evaluation as there is no strong quantitative evidence on case reduction under the different testing and typing methods. Second, our analysis also does not take into account the number of isolates required to fill a flow cell on the instrument and maximise the efficiency of sequencing runs from a laboratory perspective. In lesser populated areas, waiting for a sufficient number of isolates to complete a sequencing run or sending isolates away to other public health reference laboratories may result in a delay in the public health department receiving WGS data results, impacting the ability to implement timely public health action. While laboratory subtyping data is an important component of *Salmonella* surveillance and outbreak investigation, epidemiological and environmental investigation are also necessary for the implementation of successful interventions. We acknowledge that there are other factors in outbreaks besides laboratory typing data that may affect turn-around-time of WGS reporting. Furthermore, we assumed that WGS data will be received prospectively by the public health departments in a timely and standardized format. The harmonisation of WGS and bioinformatic methods, terminology, and reporting across public health and environmental health laboratories is essential for public health surveillance and outbreak detection nationally [28, 29]. The ability to link *Salmonella* isolates from humans, foods, water, animals, and the environment through standardized WGS data across states, regions, and even globally will be key to using WGS data effectively to prevent cases.

Our approach could be applied to other pathogens of public health relevance to ascertain how many cases of infection would need to be prevented before WGS was cost-equal to other methods. The models reflect the monetary and non-monetary costs of disease outbreaks including the opportunity costs of preventive interventions at the point of emergence. In the era of global trade and health reforms, the added value of public health extends beyond national borders and strengthens the economic imperatives for public health surveillance. Even though WGS currently has a higher cost per isolate than serotyping for *Salmonella* in 2018 in Australia, WGS will be more cost-effective if the genomic information can help prevent human infections by creating opportunities for earlier public health action and identification of vehicles of infection. While it is hard to get strong, consistent evidence to quantify case reduction using WGS, we have shown here that the effect does not need to be large for WGS to be a cheaper method. Currently WGS for public health surveillance of *Salmonella* is dependent on culture either initially or reflexively. However, there is significant research into metagenomics direct from faecal specimens [30]. Metagenomics has even been used in a limited way to investigate foodborne disease outbreaks [30]. While waiting for the costs of sequencing to decline and research in metagenomics to progress, the effective application of WGS to disease control may actually reduce costs.

## Supporting information

S1 Appendix National number of *Salmonella* spp. notifications by type, Australia, 2017. (DOCX)

S2 Appendix @Risk model. (DOCX)

S3 Appendix Outbreak scenarios. (DOCX)
