# Supplementary material for: Cost of whole genome sequencing for non-typhoidal Salmonella enterica
Source: PLoS One. 2021 Mar 19;16(3):e0248561. doi: 10.1371/journal.pone.0248561 (PMC7978342; doi:10.1371/journal.pone.0248561)
Supplement: S1 Appendix — (DOCX) [file pone.0248561.s001.docx]

**S1 Appendix: National number of *Salmonella* spp. notifications by type, Australia, 2017**

In 2017, 5687/16051 (35%) of non-typhoidal *Salmonella* notifications were serotyped as Typhimurium (Table) [1].

**Table: National number of *Salmonella* spp. notifications by type, Australia, 2017**

|  | **Number of notifications** |
| --- | --- |
| *S.* Typhimurium | 5687 |
| Non-Typhimurium *Salmonella* | 8718 |
| Unspecified | 1646 |
| Total | 16051 |

**References**

1. Department of Health. *Salmonella* public dataset [Internet]. Canberra: Australian Government; 2018 [cited 2018 21 Nov]; Available from: http://www9.health.gov.au/cda/source/pub_salmo.cfm.
