# Supplementary material for: Cost of whole genome sequencing for non-typhoidal Salmonella enterica
Source: PLoS One. 2021 Mar 19;16(3):e0248561. doi: 10.1371/journal.pone.0248561 (PMC7978342; doi:10.1371/journal.pone.0248561)
Supplement: S2 Appendix — (DOCX) [file pone.0248561.s002.docx]

**S2 Appendix: @Risk Model**

We used a novel method that allows the comparison of costs of different types of testing processes to determine the threshold of illnesses that would need to be prevented for the testing processes to be cost equal. To determine the threshold, we used the equation below in @Risk version 6 (<http://www.palisade.com>).

$$\left( {cost per test}_{A}+ cost per case \right)\times{number of cases detected with test}_{A}=$$

$$\left( {cost per test}_{B}+ cost per case \right)\times{number of cases detected with test}_{B}$$

A PERT distribution was used for variables with uncertainty. A full list of variables that were used in the different models are in the Table.

**Table: List of variables used in the @Risk model to determine the threshold of cases prevented**

| **Variable** | **Value** |
| --- | --- |
| Cost per test |  |
| PCR | $34.70 |
| Culture | $40.61 |
| Serotyping | $42.37 (range $13.82-$75.22) |
| MLVA | $52.66 (range $24.56-$95.95) |
| WGS | $83.15 (range $72.92-$95.95) |
| Cost per case | $1,098 (90% Credible Intervals $623-$1,963) |
| Cases detected with test |  |
| 2017 Total *Salmonella* notifications | 16,051 |
| 2017 *S.* Typhimurium notifications | 5,687 |
| 2017 non-Typhimurium *Salmonella* notifications | 8,718 |
| 2017 unspecified *Salmonella* notifications | 1,646 |

For example, to compare PCR-only testing to culture and WGS, the above equation is used to solve for number of cases detected with test B:

$$\left( 34.70+ PERT(623, 1098, 1963) \right)\times16051=$$

$$\left( (40361+PERT\left( 72.92, 83.15, 95.95 \right))+ PERT(623, 1098, 1963) \right)\times{number of cases detected with test}_{B}$$

The equation was run in @Risk to generate median and 90% credible intervals for the number of cases that need to be prevented.
