# Supplementary material for: Cost of whole genome sequencing for non-typhoidal Salmonella enterica
Source: PLoS One. 2021 Mar 19;16(3):e0248561. doi: 10.1371/journal.pone.0248561 (PMC7978342; doi:10.1371/journal.pone.0248561)
Supplement: S3 Appendix — (DOCX) [file pone.0248561.s003.docx]

**S3 Appendix: Outbreak scenarios**

We obtained dates of illness onset and case numbers for several outbreaks occurring over the last 20 years in Australia. We then generated epidemiological curves and cumulative outbreak costs based on current laboratory subtyping methods (serotyping and MLVA) compared with whole genome sequencing (WGS) and polymerase chain reaction (PCR) testing for a point source outbreak and two prolonged outbreaks.

Point source outbreak: *Salmonella* Typhimurium outbreak in January 2015

In this outbreak, there were 123 cases of *Salmonella* Typhimurium with illness onsets over 6 days (Figure 1). As this was an outbreak of *Salmonella* Typhimurium, serotyping and MLVA would have been used to type the isolates. As in our simulated point source outbreak, cumulative outbreak costs were slightly less if WGS had been used, costing USD 152,123 (90% CrI 92,528-257,067) for serotyping and MLVA, compared with USD 150,200 (90% CrI 90,894-255,326) for WGS or USD 139,163 (90% 79,922-244,329) for PCR-only (Figure 2).


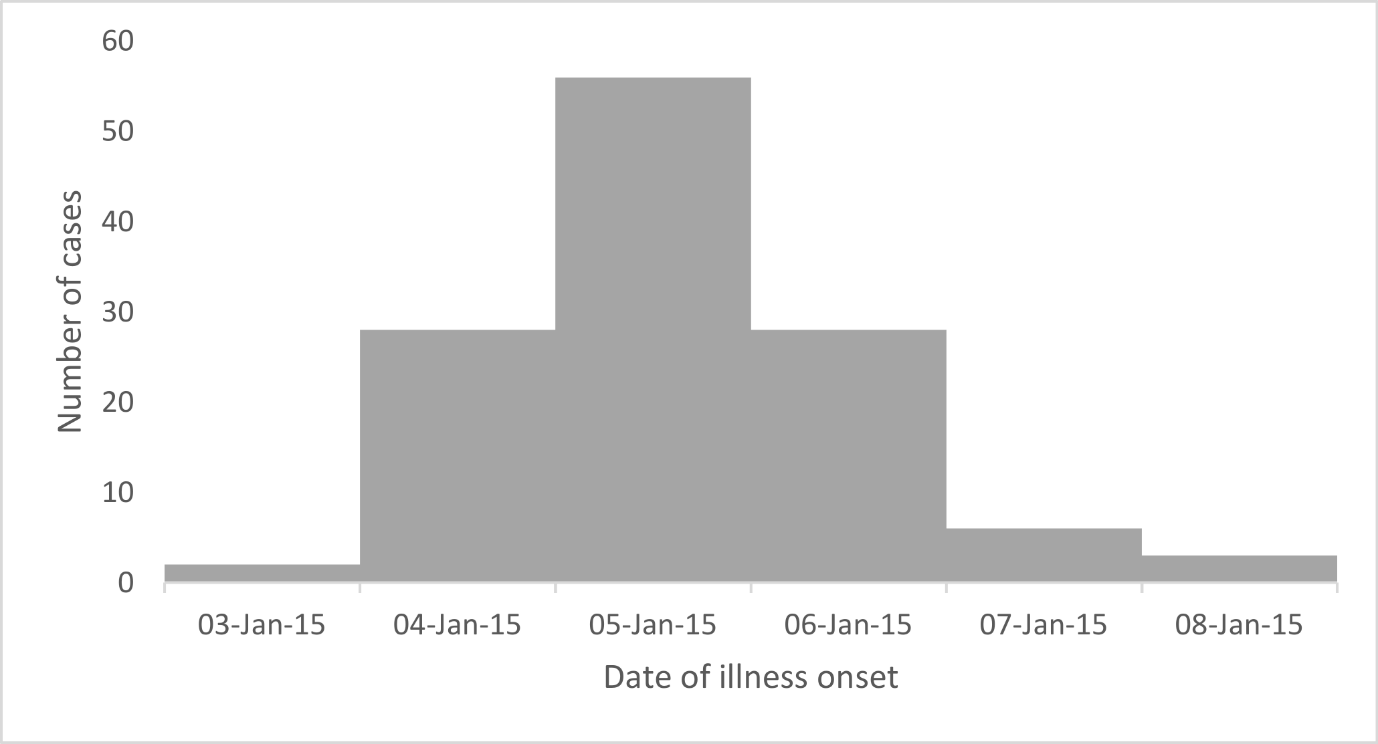


**Figure 1: Epidemiological curve of *Salmonella* Typhimurium outbreak in January 2015, Queensland Australia**


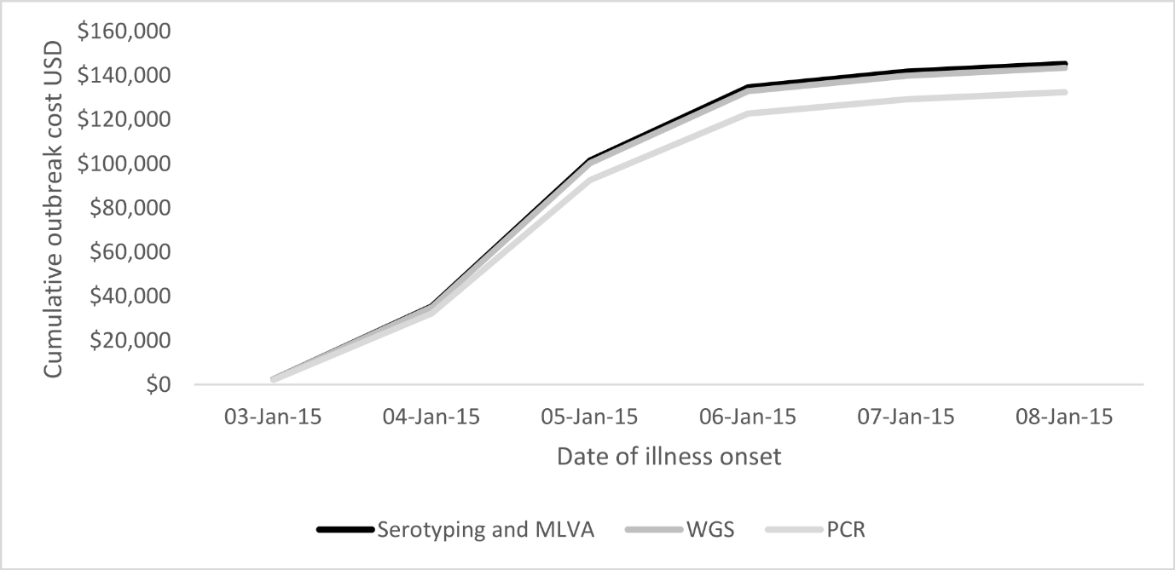


**Figure 2: Cumulative outbreak costs using serotyping and MLVA compared to WGS in a *Salmonella* Typhimurium outbreak in January 2015, Queensland Australia**

Prolonged outbreak: *Salmonella* Bovismorbificans outbreak in May and June 2001

In this outbreak, there were 29 cases of *Salmonella* Bovismorbificans with illness onsets over 44 days (Figure 3). As with the prolonged outbreak scenario, cumulative outbreak costs with WGS (USD 35,465 (90% CrI 21,504-60,616)) were higher than with serotyping (USD 34,237 (90% CrI 20,308-59,395)) or PCR (USD 32,863 (90% CrI 18,927-57,974)) (Figure 4). If WGS data were able to detect the outbreak earlier, and an intervention was put in place earlier, WGS would result in a cost savings. However, the difference in costs is not as large at our first modelled earlier intervention point (2 weeks) as was seen in our first modelled earlier intervention points in our simulated prolonged outbreak. This is due to the length of the outbreak and the shape of the epidemiological curve.


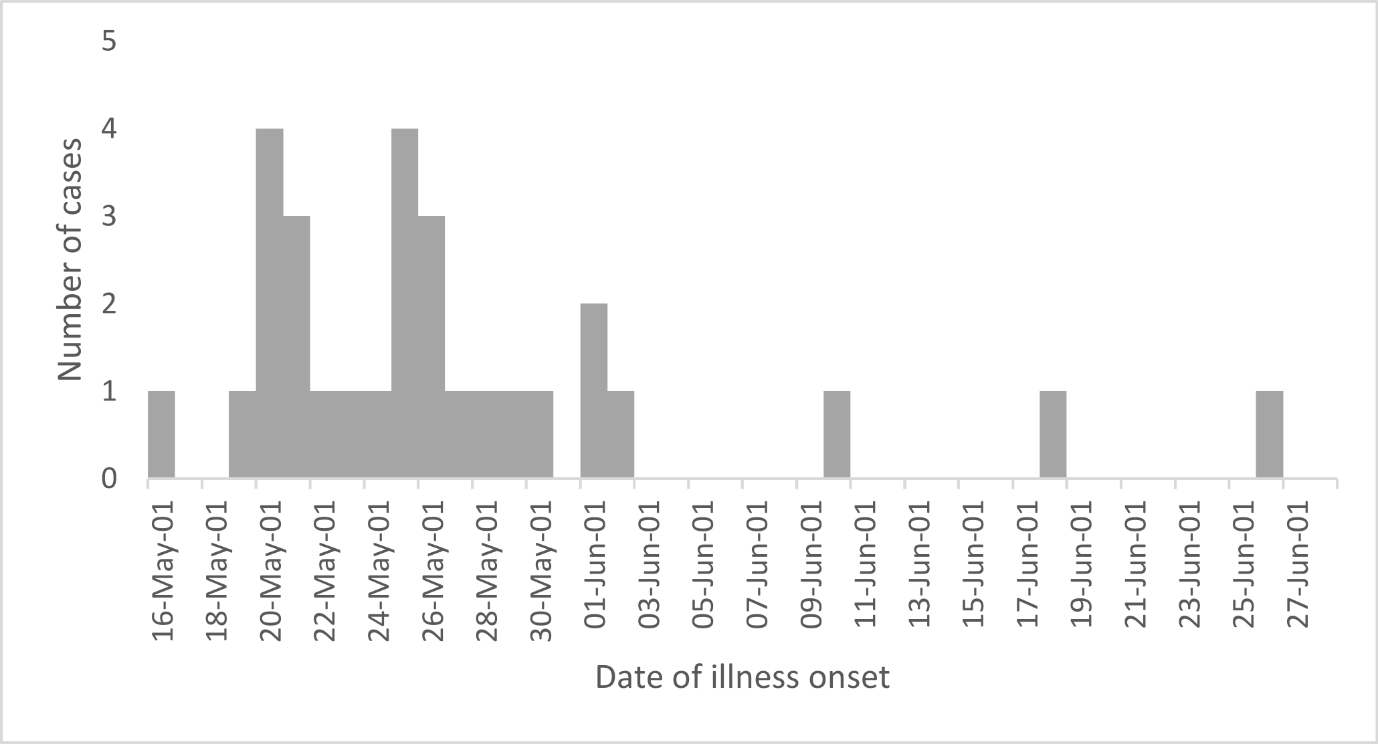


**Figure 3: Epidemiological curve of *Salmonella* Bovismorbificans outbreak in May and June 2001, Queensland Australia**


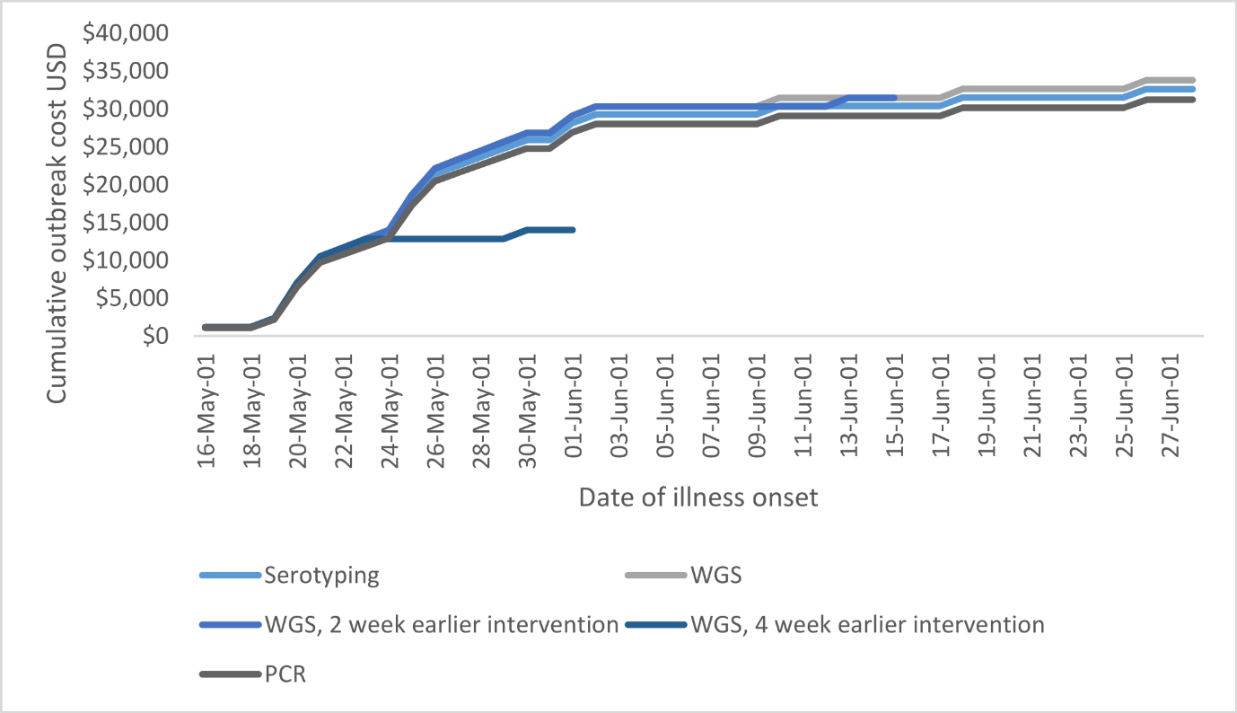


**Figure 4: Cumulative outbreak costs using serotyping compared to WGS, with earlier modelled interventions, in a *Salmonella* Bovismorbificans outbreak in May and June 2001, Queensland Australia**

As this was a *Salmonella* Bovismorbificans outbreak occurring in 2001, the isolates also were phage typed. If we added phage typing costs on top of the serotyping costs, the cumulative outbreak costs for WGS would likely be lower.

Prolonged outbreak: *Salmonella* Saintpaul outbreak in April 2011

In this outbreak, there were 17 cases of *Salmonella* Saintpaul cases occurring over 39 days (Figure 5). As with the previous example and the simulated prolonged outbreak scenario, cumulative outbreak costs with WGS (USD 20,689 (90% CrI 12,655-35,477) were higher than with serotyping (USD 20,011 (90% CrI 11,969-34,824)) or PCR (USD 19,159 (90% CrI 11,133-33,982)) (Figure 6). If an intervention could be put into place and prevent just one case, then WGS would be cheaper.


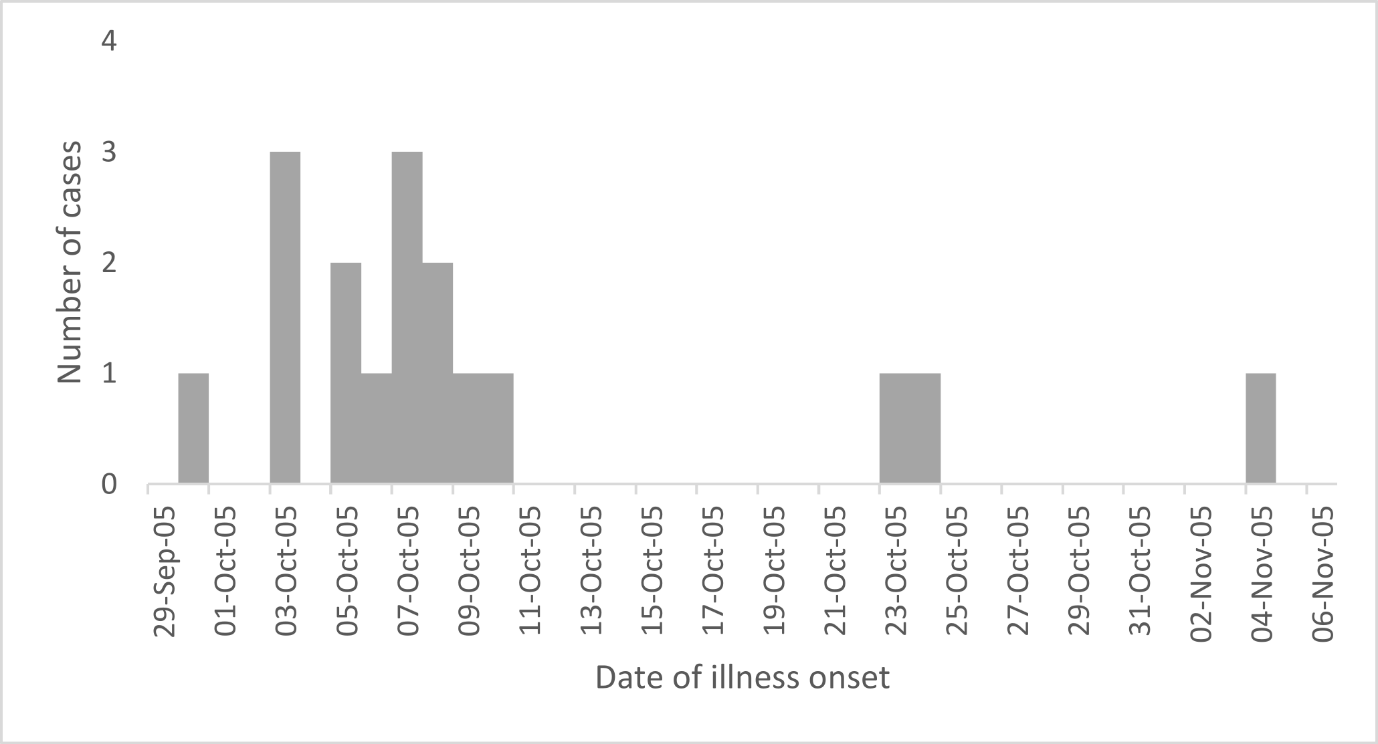


**Figure 5: Epidemiological curve of *Salmonella* Saintpaul outbreak September–November 2005, Queensland Australia**


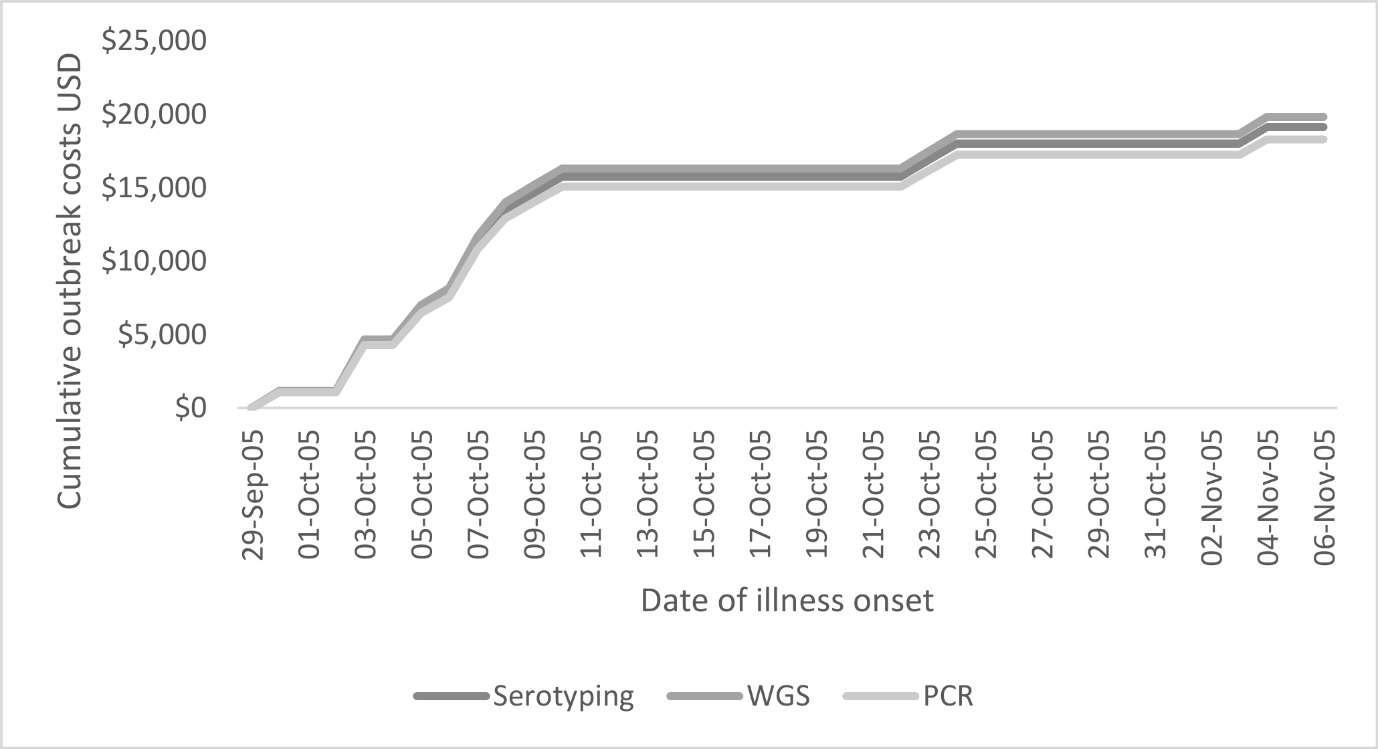


**Figure 6: Cumulative outbreak costs using serotyping compared to WGS in a *Salmonella* Saintpaul outbreak in 2005, Queensland Australia**
